# Supplementary material for: Resolving the full spectrum of human genome variation using Linked-Reads
Source: Genome Res. 2019 Apr;29(4):635–45. doi: 10.1101/gr.234443.118 (PMC6442396; doi:10.1101/gr.234443.118)
Supplement: Supplemental Material [file supp_gr.234443.118_Supplemental_longranger-2.2.zip › longranger-2.2.tar.bz2!/lr2.2-public/lib/stan/cmdstan/stan/doc/api/html/agrad_2rev_2matrix_8hpp_source.html]

Stan: src/stan/agrad/rev/matrix.hpp Source File


|  |  |
| --- | --- |
|  | Stan  2.5.0  probability, sampling & optimization |


- Main Page
- Related Pages
- Namespaces
- Classes
- Files

- File List
- File Members

All Classes Namespaces Files Functions Variables Typedefs Enumerations Enumerator Friends Macros Pages

- src
- stan
- agrad
- rev

matrix.hpp

Go to the documentation of this file.

1 #ifndef STAN\_\_AGRAD\_\_REV\_\_MATRIX\_HPP

2 #define STAN\_\_AGRAD\_\_REV\_\_MATRIX\_HPP

3

4 #include <stan/agrad/rev/matrix/Eigen\_NumTraits.hpp>

5 #include <stan/agrad/rev/matrix/LDLT\_alloc.hpp>

6 #include <stan/agrad/rev/matrix/LDLT\_factor.hpp>

7 #include <stan/agrad/rev/matrix/crossprod.hpp>

8 #include <stan/agrad/rev/matrix/determinant.hpp>

9 #include <stan/agrad/rev/matrix/divide.hpp>

10 #include <stan/agrad/rev/matrix/dot\_product.hpp>

11 #include <stan/agrad/rev/matrix/dot\_self.hpp>

12 #include <stan/agrad/rev/matrix/grad.hpp>

13 #include <stan/agrad/rev/matrix/initialize\_variable.hpp>

14 #include <stan/agrad/rev/matrix/log\_determinant.hpp>

15 #include <stan/agrad/rev/matrix/log\_determinant\_ldlt.hpp>

16 #include <stan/agrad/rev/matrix/log\_determinant\_spd.hpp>

17 #include <stan/agrad/rev/matrix/log\_softmax.hpp>

18 #include <stan/agrad/rev/matrix/log\_sum\_exp.hpp>

19 #include <stan/agrad/rev/matrix/mdivide\_left.hpp>

20 #include <stan/agrad/rev/matrix/mdivide\_left\_ldlt.hpp>

21 #include <stan/agrad/rev/matrix/mdivide\_left\_spd.hpp>

22 #include <stan/agrad/rev/matrix/mdivide\_left\_tri.hpp>

23 #include <stan/agrad/rev/matrix/multiply.hpp>

24 #include <stan/agrad/rev/matrix/multiply\_lower\_tri\_self\_transpose.hpp>

25 #include <stan/agrad/rev/matrix/quad\_form.hpp>

26 #include <stan/agrad/rev/matrix/sd.hpp>

27 #include <stan/agrad/rev/matrix/softmax.hpp>

28 #include <stan/agrad/rev/matrix/sort.hpp>

29 #include <stan/agrad/rev/matrix/squared\_distance.hpp>

30 #include <stan/agrad/rev/matrix/stan\_print.hpp>

31 #include <stan/agrad/rev/matrix/stored\_gradient\_vari.hpp>

32 #include <stan/agrad/rev/matrix/sum.hpp>

33 #include <stan/agrad/rev/matrix/tcrossprod.hpp>

34 #include <stan/agrad/rev/matrix/to\_var.hpp>

35 #include <stan/agrad/rev/matrix/trace\_gen\_inv\_quad\_form\_ldlt.hpp>

36 #include <stan/agrad/rev/matrix/trace\_gen\_quad\_form.hpp>

37 #include <stan/agrad/rev/matrix/trace\_inv\_quad\_form\_ldlt.hpp>

38 #include <stan/agrad/rev/matrix/trace\_quad\_form.hpp>

39 #include <stan/agrad/rev/matrix/typedefs.hpp>

40 #include <stan/agrad/rev/matrix/value\_of.hpp>

41 #include <stan/agrad/rev/matrix/variance.hpp>

42

43 #endif

log\_determinant\_ldlt.hpp

determinant.hpp

LDLT\_alloc.hpp

quad\_form.hpp

log\_softmax.hpp

softmax.hpp

typedefs.hpp

crossprod.hpp

Eigen\_NumTraits.hpp

log\_determinant.hpp

grad.hpp

initialize\_variable.hpp

log\_determinant\_spd.hpp

log\_sum\_exp.hpp

sort.hpp

sd.hpp

dot\_self.hpp

variance.hpp

mdivide\_left\_ldlt.hpp

to\_var.hpp

sum.hpp

dot\_product.hpp

squared\_distance.hpp

value\_of.hpp

trace\_gen\_inv\_quad\_form\_ldlt.hpp

multiply\_lower\_tri\_self\_transpose.hpp

mdivide\_left\_spd.hpp

trace\_inv\_quad\_form\_ldlt.hpp

stored\_gradient\_vari.hpp

multiply.hpp

divide.hpp

trace\_quad\_form.hpp

tcrossprod.hpp

stan\_print.hpp

mdivide\_left\_tri.hpp

trace\_gen\_quad\_form.hpp

mdivide\_left.hpp

LDLT\_factor.hpp


---

[ Stan Home Page ]

*© 2011–2014,
Stan Development Team.*
